# How Rainfall Variation Influences Reproductive Patterns of African Savanna Ungulates in an Equatorial Region Where Photoperiod Variation Is Absent

**DOI:** 10.1371/journal.pone.0133744

**Published:** 2015-08-21

**Authors:** Joseph O. Ogutu, Norman Owen-Smith, Hans-Peter Piepho, Holly T. Dublin

**Affiliations:** 1 International Livestock Research Institute, P. O. Box 30709, Nairobi, 00100, Kenya; 2 University of Hohenheim, Institute for Crop Science, 70599 Stuttgart, Germany; 3 Centre for African Ecology, School of Animal, Plant and Environmental Sciences, University of the Witwatersrand, Wits, 2050, South Africa; 4 IUCN ESARO, Wasaa Conservation Centre, P.O. Box 68200, Nairobi, Kenya, 00200; University of Sydney, AUSTRALIA

## Abstract

In high temperate latitudes, ungulates generally give birth within a narrow time window when conditions are optimal for offspring survival in spring or early summer, and use changing photoperiod to time conceptions so as to anticipate these conditions. However, in low tropical latitudes day length variation is minimal, and rainfall variation makes the seasonal cycle less predictable. Nevertheless, several ungulate species retain narrow birth peaks under such conditions, while others show births spread quite widely through the year. We investigated how within-year and between-year variation in rainfall influenced the reproductive timing of four ungulate species showing these contrasting patterns in the Masai Mara region of Kenya. All four species exhibited birth peaks during the putative optimal period in the early wet season. For hartebeest and impala, the birth peak was diffuse and offspring were born throughout the year. In contrast, topi and warthog showed a narrow seasonal concentration of births, with conceptions suppressed once monthly rainfall fell below a threshold level. High rainfall in the previous season and high early rains in the current year enhanced survival into the juvenile stage for all the species except impala. Our findings reveal how rainfall variation affecting grass growth and hence herbivore nutrition can govern the reproductive phenology of ungulates in tropical latitudes where day length variation is minimal. The underlying mechanism seems to be the suppression of conceptions once nutritional gains become insufficient. Through responding proximally to within-year variation in rainfall, tropical savanna ungulates are less likely to be affected adversely by the consequences of global warming for vegetation phenology than northern ungulates showing more rigid photoperiodic control over reproductive timing.

## Introduction

Large mammalian herbivores depend on a food resource that varies seasonally in amount and quality because of corresponding seasonal patterns of growth and dormancy shown by plants [1]. Accordingly, their births are typically concentrated early in the growing season when food quality is highest [2], thereby supporting the peak nutritional demands of mothers through late pregnancy and early lactation [3]. For medium- to—large ungulates, the corresponding conception peak falls during late summer or early autumn. In north temperate latitudes, the scheduling of oestrous cycling and hence conceptions is tightly governed by daily variation in the photoperiod, and births are narrowly concentrated within a 2–3 week window [4]. Observations on animals held in zoos show that many tropical and subtropical ungulates also respond to changing day length in their reproductive phenology [4,5]. However, near the equator changing photoperiod is no longer a tenable cue for the timing of mating and hence conceptions. In these circumstances, many ungulate species reproduce year-round [6,7,8]. Nevertheless, certain ungulate species retain a seasonal birth pulse even in the absence of much day length variation, suggesting that other factors govern their reproductive phenology. Predation on newborn offspring could contribute to narrowing the birth peak [9,10], but does not explain the timing of births within the seasonal cycle.

For African elephants (*Loxodonta africana*), births tend to be concentrated early in the wet season, and result from conceptions occurring also during the wet season, following a gestation period of 22 months [11,12]. However, for rhinos (*Ceratotherium simum* and *Diceros bicornis*) and giraffe (*Giraffa camelopardalis*), conceptions peaking during the wet season generate a birth pulse early in the dry season, because for these species the gestation period spans 15-16-months [13,14]. This suggests proximate control by conditions affecting oestrus and mating rather than by the food quality around the time of births. In equatorial regions the ultimate benefits from nutritional conditions during the time of peak demands cannot be anticipated from the cue provided by changing day length. This raises the possibility that the reproductive phenology of medium-sized ungulates could likewise become governed proximately by seasonal variation in rainfall affecting the timing of conceptions where photoperiod cues are lacking.

The reproductive performance of ungulates in high northern latitudes appears to be threatened by the effects of global warming on the timing of plant growth, disrupting the synchrony between births and optimal nutritional conditions for late pregnancy and lactation [15]. The amplified variation in rainfall anticipated as a consequence of global warming [16] could also be adverse for herbivores occupying tropical savanna ecosystems by disrupting seasonal nutritional regimes. While low rainfall restricts plant growth and hence reduces the nutritional value of plant parts, too much rainfall could also be detrimental by promoting the growth of taller grass higher in fibre contents. Accordingly, our aim in this paper is to assess how annual and seasonal variation in rainfall affects the reproductive schedules and performance of ungulates inhabiting an equatorial region. Our analysis compares two ungulate species that retain seasonally restricted births even in equatorial East Africa with two that produce offspring year-round despite showing narrow birth peaks at higher southern latitudes. Rainfall variation could potentially influence reproductive schedules and performance by (1) affecting recovery in body condition from the previous pregnancy towards the threshold enabling oestrous cycling; (2) influencing foetal growth and viability; (3) controlling the post-birth growth and survival of offspring. Accordingly, our specific hypotheses were:- H_1_: The fertility of female ungulates reproducing year-round should be less responsive to rainfall than species showing narrow birth pulses. H_2_: Monthly fertility should depend on rainfall over some period immediately prior to the time of conception if nutrition directly influences oestrous cycling and establishment of the foetus. H_3_: Annual fecundity should depend on rainfall received through some extended period prior to conceptions if body condition influences the proportion of females that are able to conceive and support the foetus. H_4_: Survival from birth into the juvenile stage should depend on preceding dry season rainfall affecting foetal growth and hence birth mass, and on early wet season rainfall affecting post-natal growth.

## Materials and Methods

### Study area

Our study area was the Masai Mara National Reserve, covering 1530 km² in south-western Kenya (1°13′-1°45′S, 34°45′-35°25′E). Rainfall can be partitioned between the early “short” rains (Nov-Jan), and the later “long” rains (Mar-Jun), typically separated by a mild lull in February [17]. Rainfall was recorded over a network of 14 monthly storage and two daily gauges. The annual rainfall total over 1965–2003 averaged 1010 ± 187 mm, including 785 ± 152 mm during the eight wet season months and 214 ± 76 mm during the four dry season months. Monthly temperatures varied little between a mean daily maximum of 24.8°C in February and a mean daily minimum of 9.8°C in September. Grazing pressure keeps the grass standing crop relatively low through October-February, with peak biomass reached in June [18]. Leaf concentrations of nitrogen start rising during September to a peak in December, and thereafter decline as grass height increases and above-ground parts senesce through the long rains into the dry season [19].

### Ethics Statement

Permission to conduct the monitoring was granted by the Office of the President of the Republic of Kenya, the Narok County Government (formerly Narok County Council), the Kenya Wildlife Service (KWS), Wardens of the Masai Mara National Reserve and the management of the former Koyiaki Group Ranch.

#### Population surveys and demography

Monthly vehicle counts of resident ungulate species were organised by the Masai Mara Ecological Monitoring Program from July 1989 to December 2003. We used only the data for the years with complete breeding cycles spanning 1989 to 2002. The study area was subdivided into three census blocks using major rivers and roads, each with a fixed transect [20,21]. During the 174-month monitoring period, counts were missing for 17 months distributed over nine years, for a further five months on one transect, and for one month on another transect. In this paper, we compare the reproductive patterns of topi (*Damaliscus lunatus korrigum*) and warthog (*Phacochoerus africanus*), which showed seasonally restricted births, with hartebeest (*Alcelaphus busephalus*) and impala (*Aepycerus melampus*), which give birth year-round in East Africa (Fig A in S1 File), despite reproducing seasonally in southern Africa. Topi and hartebeest have gestation periods of around 8 months, while the gestation period of impala is 6.5 months, and that of warthog 5.5 months [4,22].

A combination of body size, coat colour, horn length and shape was used to assign immature animals to five size classes; newborn, quarter, half, yearling and three quarter grown. The newborn class represents animals judged to be under one month old. For analysis quarter and half-grown animals were grouped as juveniles for impala and warthog. Animals were sexed using the presence, size and shape of horns and other secondary sexual characters when present, except for warthog which lack sufficient sexual dimorphism. Animals were highly visible in the open grasslands, reducing misclassification into these age-sex classes. To reduce the omission of potentially fecund young females, three-quarter-sized hartebeest and topi and yearling plus three-quarter-sized impala and warthog were included in the adult class. The monthly fecundity for each species was estimated by dividing the total number of newborns recorded in each month by the corresponding number of adult females. In the case of warthog we divided the total number of newborns by half the number of all adults to approximate the proportion of the adults constituted by females.

However, when calculating the effective fertility dependent on prior rainfall we needed to allow for females that were already pregnant, and thus not able to conceive. The pregnant proportion was calculated by summing monthly fecundities back over the gestation period. The effective monthly fertility in each month was calculated as the number of newborns divided by the number of females excluding the pregnant proportion. Total annual fecundity was obtained by summing monthly fecundities over the annual cycle (July-June). This sum should not exceed 1 but may do so due to errors in age estimation within the newborn category. Age estimation for the newborn category is much more difficult for hartebeest than for topi because the young of hartebeest are not born in a narrow pulse like those of topi. The estimated age for the newborn class is thus likely to be biased and the magnitude of this bias is expected to be larger for hartebeest than for topi. To reduce such biases, we multiplied the summed proportion of newborns by 0.8 for topi and 0.67 for hartebeest, allowing for the differences expected in the relative biases for the two species. This adjustment ensured that the summed proportions for newborn topi and hartebeest did not exceed 1 because both species only give birth to a single young per year. No correction was needed for impala, and some estimates of annual fecundity were allowed to exceed 1 because of the possibility that some females could give birth twice during the course of a year. For warthog we multiplied the summed proportion of newly born piglets per adult by 0.5 to account for multiple offspring per litter and by a further 0.5 to approximate the sex ratio of adults. This adjustment is a rough approximation because we did not know the actual mean litter size for our study area. The various adjustments that we made to counteract biases do not affect statistical relationships because it is the relative value of the fertility and annual fecundity estimates that matter, not their absolute values. For the two seasonally breeding species, survival from birth into the juvenile stage was calculated by relating the mean proportion of juveniles over three months following the end of the birth peak to the summed proportion of newborns through the peak. Correspondingly, to estimate survival from juvenile into yearling stage, the mean proportion of juveniles aged around 3 months was related to the mean proportion of yearlings aged around 12 months after the birth peak. For the two non-seasonal species, merely the proportions of newborns, juveniles and yearlings relative to adult females averaged over blocks of three months were derived. For certain analyses, fertility was assessed over periods representing early births, summed over August-October, modal births, summed over November-December, and late births, summed through January-March.

### Statistical analyses

#### Relationships with seasonal or annual blocks of rainfall

Relationships of monthly fertility, annual fecundity and offspring survival with prior blocks of rainfall were assessed by linear regression analyses.

Effective monthly fertility was related to monthly rainfall averaged over a time window spanning the month of conception identified by cross-correlation analysis and the distributed lag nonlinear model [8]. The effective monthly fecundity was grouped by the season (early wet, late wet and dry seasons) in which conception occurred to test the hypothesis that rainfall influences the likelihood of conceptions. We dummy coded the seasons of conception to permit relating effective monthly fecundity to rainfall using possibly different functional relationships (linear or quadratic terms in rainfall) for each season of conception in the same model. We expanded the model by adding the wet season rainfall in the preceding year, the current dry season rainfall or both rainfall components to the model with rainfall block and season of conception as the only predictors but this did not improve the fit for any of the four species.

Biologically, very low rainfall post-conception may cause foetal losses and hence fewer births. Because this mechanism is distinct from conception, we considered different functional relationships with fecundity emanating from the rainfall blocks. Moreover, since the effect of rainfall post-conception is contingent on conceptions, it cannot be assessed independently, but rather as a modifier of the births that would otherwise have been generated by rainfall prior to conception. As a result, we considered the separate contributions of rainfall for the block of months pre-conception and block of months post conception, subdividing the overall best-fit block accordingly. Specifically, we used rainfall averaged over lags 9–12 or 9–11 and 7–8 to represent rainfall influences pre-conception and post-conception, respectively, for topi and hartebeest with gestation lengths of 7.5–8 months. The corresponding rainfall blocks were averages over lags 7–10 and 6–7 for warthog and impala with gestation lengths of 5.5–6 months. Dummy coding of conception seasons was similarly used to enable relating monthly apparent fecundity to each of these rainfall blocks. The data sets used in all the statistical analyses and plots are provided in (S1–S3 Datas). The complete monthly age- and sex-structured counts of the four study species and four additional species (giraffe, ostrich *Struthio camelus massaicus*, waterbuck *Kobus ellipsiprymnus* and zebra *Equus quagga*) covering July 1989 to December 2003 are provided in (S4 Data). The rainfall data for the study area for 1965–2004 averaged over all the rain gauges are provided in (S5 Data).

## Results

### Monthly fertility and prior rainfall

**Fig 1:**
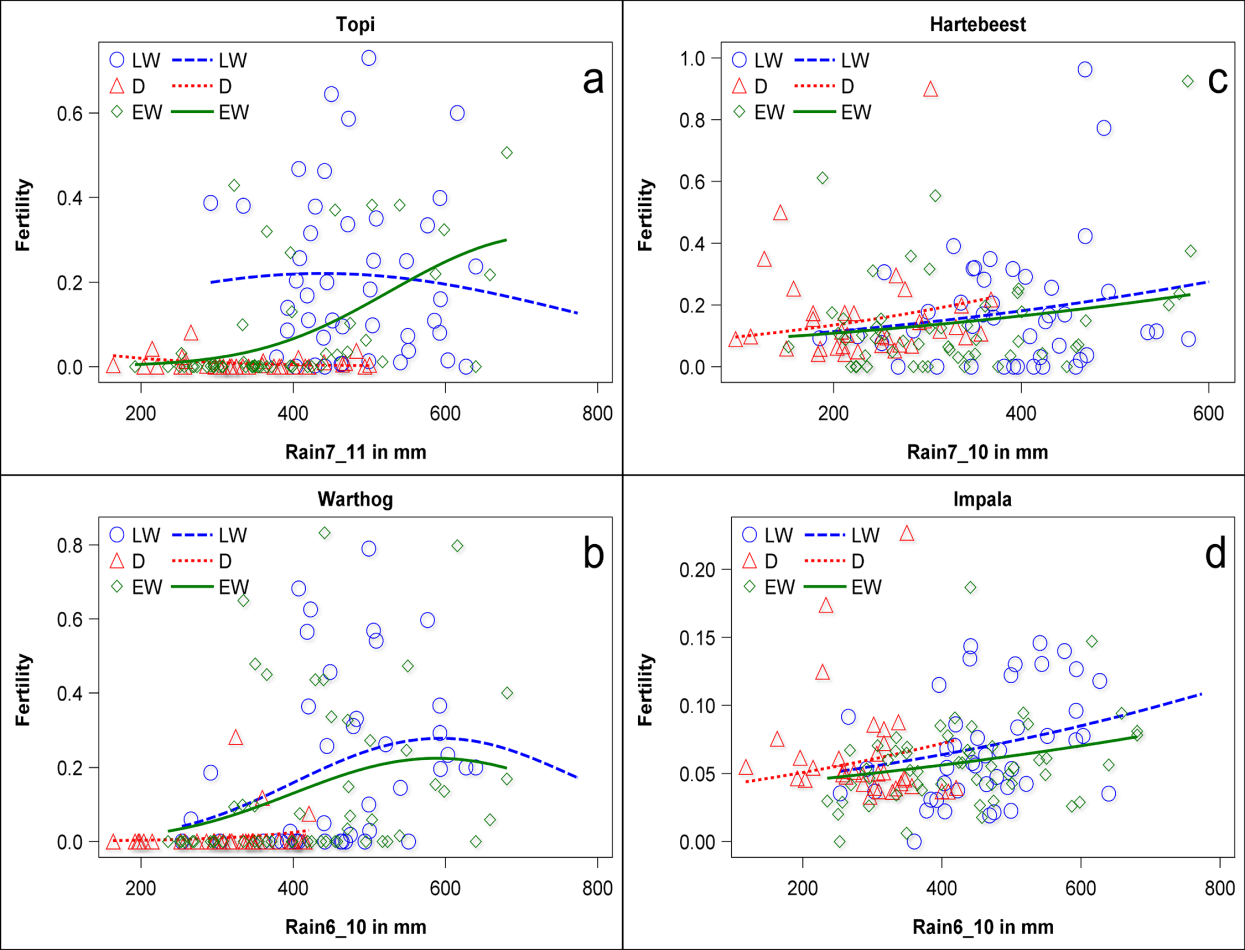
Relationships between apparent fecundity and the best-supported rainfall block spanning conception months for each ungulate species for the early wet (Nov-Dec), late wet (Jan-June) and dry (Jul-Oct) seasons of conceptions for a) topi, b) warthog, c) hartebeest and d) impala.

For topi, effective monthly fertility was mostly strongly related to the rainfall block 7–11 mo prior to births, although this period spanned post-conception as well as pre-conception months (Fig 1A, S1 and S2 Tables). However, the form of the relationship depended on the seasonal period during which conceptions occurred. Early season fertility showed an accelerating increase with rainfall prior to conception, while late season fertility showed no relationship with generally high prior rainfall. Very few conceptions occurred during the dry season months, when most females were already pregnant. Across all seasonal periods, fertility remained low unless prior rainfall had exceeded about 250 mm summed over 4 months. A reduction of fertility by low rainfall post-conception was supported statistically only for late wet or dry season conceptions (S2 Table). Although high prior rainfall also appeared to depress late conceptions, this was largely because the highest rainfall was associated with conceptions occurring as late as Jan-Feb, after the seasonal peak. Rainfall in the month of conception had a stronger effect on effective monthly fertility than rainfall prior to conception, suggesting that the forage quality prevailing at the time of conception influences whether the foetus survives in the first month.

For warthog, the strongest statistical relationship was with rainfall 6–10 months prior to births, in this case spanning only the pre-conception period. Patterns largely replicated those shown by topi, except for an apparent depressant effect of high prior rainfall on late season conceptions (Fig 1B, S1 and S2 Tables). Few warthog conceived when prior rainfall had been under about 300 mm. For hartebeest, there was a very weak although significant relationship between monthly fertility and rainfall 7–10 mo prior to births, spanning the conception period (Fig 1C). Separate relationships with rainfall blocks pre- and post-conception were not apparent (S1 and S2 Tables), and conceptions frequently occurred even when prior rainfall had totalled <200 mm over the preceding 4 mo. Impala showed a very similar pattern to hartebeest (Fig 1D, S1 and S2 Tables).

### Fecundity and survival relationships with seasonal or annual blocks of rainfall

**Fig 2:**
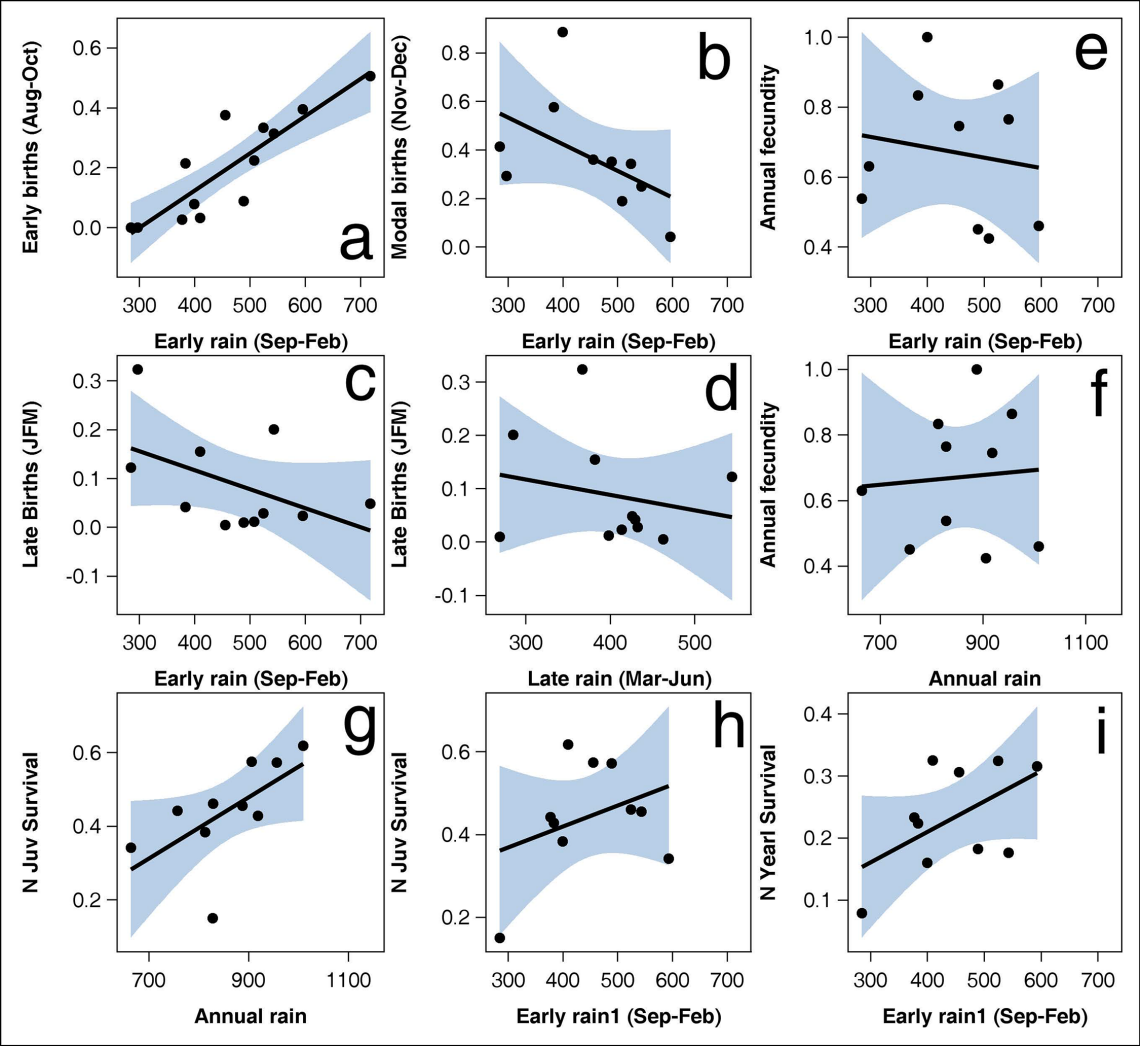
Relationships between a) early births (August-October), b) modal births (November-December), c) late births (January-March) and e) annual fecundity and early rainfall (September-February) in the current year; d) late births and late rainfall (March-June), f) annual fecundity and g) juvenile survival and annual rainfall (Nov-Jun) in the current year; h) juvenile survival and i) yearling survival and early rainfall in the preceding year for topi. The patterns for topi well illustrate those for the other species.

For topi, early season births (Aug-Oct) were positively related to early season rainfall (Sep-Feb) prior to conception (Fig 2A). Consequently, modal (Nov-Dec) and late (Jan-Mar) births appeared to be negatively related to early season rainfall (Fig 2B and 2C), while late births showed no relation with late season rainfall (Mar-Jun) preceding the time of conception (Fig 2D, S3 Table). For warthog the relationship between early births and early season rain was positive but much weaker than for topi (Fig A in S2 File, S3 Table). For hartebeest and impala, early season births were unrelated to early season rainfall. Modal or late season births and annual fecundity appeared to be negatively related to seasonal or total annual rainfall for these two species, as for topi (Fig A in S3 and S4 Files, S3 Table).

Total annual fecundity was related neither to early season rainfall nor to the total annual rainfall for female topi (Fig 2E and 2F, S3 Table). However, offspring survival into the juvenile stage was positively related to the preceding annual rainfall total (Fig 2G) and, less strongly, to the current early season rainfall (Fig 2H). Further survival into the yearling stage appeared similarly dependent on the early season rainfall in the current year (Fig 2I). For warthog there were indications of humped relationships with prior rainfall especially for survival into the juvenile stage (Fig A in S2 File). Hartebeest and impala both showed negative relationships between annual fecundity and early season rainfall, although little or no relationship was evident with the annual rainfall total (Fig A in S3 and S4 Files). For impala the neonate proportion during the early season appeared positively related to early rains preceding conception. No other significant relationships with rainfall were evident either for impala or for hartebeest (S3 Table). Survival into the juvenile stage increased linearly with increases in the preceding dry season rainfall during gestation only for warthog (*F*_1,7_ = 6.14, *P* = 0.0423) but was not significantly correlated with the early wet season rainfall post-birth for all the four species.

## Discussion

Our results reveal two distinct patterns, one represented by the two ungulate species that show clear seasonal peaks in births, and the other by the two ungulate species with births widely spread throughout the year. For topi and warthog, very few females conceived unless prior rainfall over the preceding block of 4–5 months had exceeded a threshold value of around 250–300 mm. For hartebeest and impala, the effect of rainfall on the effective fertility was very weak, with the result that conceptions continued to occur during the dry season months despite little prior rainfall. Correspondingly, more female topi conceived earlier in years when preceding rainfall had been higher, while warthog showed a similar trend, albeit less strongly. In contrast, hartebeest and impala showed no effect of prior rainfall on the proportion of females conceiving during this period. Topi and warthog showed a consistently positive relationship between prior annual rainfall and offspring survival after birth, but prior rainfall had no effect on the survival of newborn hartebeest and impala. In general hartebeest and impala were unresponsive to rainfall variation during the course of the year in their reproductive performance, in contrast to topi and warthog. The observation that the rainfall influence extended through the conception month for topi suggests that foetal loss post-conception in response to low rainfall around this critical time might contribute to the effective fertility that we calculated. We found only weak indications that exceptionally high rainfall inhibited conceptions and hence subsequent births by producing excessively fibrous forage.

The distinction in reproductive phenology between topi and hartebeest was surprising, considering that they are allied phylogenetically in the same antelope tribe (Alcelaphini) and seem at least superficially similar in their grass dependency. However, the distribution of hartebeest extends into drier regions of southern Africa than that of tsessebe (*D*.*l*. *lunatus*, conspecific with topi). In southern Africa, both these antelope species show seasonal birth peaks timed early during October-November, around the start of the rains [23–26]. Topi likewise breed seasonally in the Tanzanian section of the Serengeti ecosystem (1° to 3° S), but with peak births occurring a month earlier than in Mara, consistent with the earlier start of the short rains in the Serengeti. Hartebeest reproduce year-round in Serengeti, with weak bimodal peaks in Aug-Sep and Dec, similar to the pattern in Mara. Wildebeest (*Connochaetes taurinus*) in Serengeti exhibit a narrow concentration of births during February-March when the migrants throng the short grass plains. Their conceptions evidently do not respond to the early season rains experienced while they are still migrating southwards. Instead, conceptions are deferred to the early dry season after the initiation of the return migration northwards. Zebra show a peak in births during the late rains in both Serengeti and southern Africa, although this is somewhat diffuse, probably because their 12-month gestation disrupts the seasonal synchrony of successive births.

Warthog show a narrow seasonal concentration of births in Serengeti as well as in southern Africa. However, warthog give birth in all months where there is sufficient rainfall year-round (e.g. in western Uganda, Zaire and Congo Brazzaville, [27,28]. Impala reproduce year-round in Serengeti, with weak bimodal peaks in births in Jun-Aug and Oct-Jan [6]. In southern Africa south of the Zambezi River, impala consistently show a narrow birth peak, with 80% of lambs born within a 2-week window extending from late November into early December [5,25,29,30].

Variability in reproductive seasonality is also evident among other ungulate species. Although sable antelope (*Hippotragus niger*) show a birth peak spread over about two months in southern Africa, the timing of this peak varies from January-March in South Africa [23], Botswana [31] and Zimbabwe [32,33] to June-September in Zambia [34, 35] and Angola [36]. Near the equator in Kenya sable breed throughout the year [37]. Sable reproduce year-round in zoos, indicating a lack of photoperiodic control. However, roan antelope (*Hippotragus equinus*), which likewise lack responsiveness to photoperiod in zoos [4], produce calves through most months of the year in both eastern and southern Africa [38], as also do waterbuck [6,39,40]. Other species giving birth throughout the year in southern Africa include bushbuck (*Tragelaphus scriptus*) and nyala (*Tragelaphus angasi*), dependent largely on browse with a different seasonal pattern of growth to grasses [26]. Gemsbok (*Oryx gazella*), which are grazers inhabiting arid savanna regions where rainfall patterns are erratic, reproduce in all months of the year [5].

Year-round reproduction is the null response expected in equatorial latitudes where day length variation is minimal, subject to minor variation from prevailing nutrition. This is the pattern typical of impala, hartebeest and certain other ungulate species in equatorial East Africa. In tropical India where monsoon rainfall generates seasonality in forage availability and quality, chital (*Axis axis*) give birth early in the wet season when forage quality is highest, while the much larger gaur (*Bos gaurus*) produces offspring throughout the year [41]. Apparently gaur are able to satisfy their minimum forage requirements all year round, while lactating chital do so for less than 40% of the year [41]. The antelope that breed year-round in southern Africa are either grazers associated with habitats retaining some green grass year-round (e.g., waterbuck typically occur near water, while roan antelope favour grassy dambos or vleis [42], browsers like bushbuck, or occupy arid environments where the seasonal growth of grass is unpredictable from year to year, like gemsbok.

For megaherbivores, sensitive stages of reproduction are spread through the year as a consequence of prolonged gestation and slow growth to maturity of offspring. Despite this, megaherbivores show distinct peaks in births resulting from a rise in conceptions during the early rains [2,14]. Hence oestrous cycling in these species seems to be influenced proximally by the prevailing nutritional regime, rather than governed ultimately by the conditions anticipated around the time of births. The underlying mechanism appears to be the suppression of oestrous cycling by low or deteriorating food quality [2,14,43]. A similar mechanism might operate for topi and warthog, which in Mara rarely conceived when prior rainfall had totalled less than 300 mm over the preceding few months. As a consequence of the dry season suppression, a surge in conceptions occurs once early season rains alleviate this constraint, delayed by the period required by females to recover their body condition. Accordingly, conceptions become shifted earlier in years with higher early season rains, as previously reported by [7,44], but without much effect on annual fecundity due to seasonal compensation. Annual variation in recruitment was governed mainly by offspring viability through the early post-birth period, rather than by the effects of rainfall on fertility. Hartebeest did not show much seasonal reduction in fertility, perhaps because they are less strongly dependent on green grass than topi, as implied by their wider distribution into drier regions. Impala are less responsive to rainfall because they can obtain green browse through the dry season.

A further influence on seasonal reproductive patterns could be whether offspring hide or follow their mothers shortly after birth. For followers a narrowing of the birth peak is favoured by predator swamping [9], while for hiders this mechanism is unimportant. Topi calves are followers and warthog piglets follow the mother after emerging from their burrows while still highly vulnerable to predators. In contrast, hartebeest calves lie out for an extended period and impala lambs for at least several days [22,45,46].

Nevertheless, the minimum monthly rainfall of around 50 mm during the dry season in Masai Mara is vastly higher than the dry season rainfall experienced in southern African savannas, where zero rainfall may be recorded during several successive months. This raises the possibility that the seasonally restricted reproduction typical of many ungulate species in southern Africa might be an outcome of the nutritional suppression of oestrus during the acutely dry season, rather than governed by photoperiodic cues. This could help explain the unusually early birth peaks shown by tsessebe, hartebeest and warthog in southern Africa. Other grazers, such as wildebeest, have birth peaks better synchronized with the availability of high quality forage, and corresponding mating peaks scheduled following the end of the wet season.

In conclusion, our findings highlight the influences of seasonal and annual variation in rainfall on the reproductive phenology of tropical savanna ungulates. Species responding flexibly to variable rainfall patterns are less likely to be threatened by reproductive mismatch due to the effects of global climate change on plant phenology than ungulates inhabiting high northern latitudes where day length more rigidly controls the timing of births. Nevertheless, widened annual variation in rainfall could threaten populations of savanna herbivores by affecting postnatal survival.

## Supporting Information

S1 Data Effective fertility data. (XLSX)

S2 Data Effective fertility data corrected for pregnancy. (XLSX)

S3 Data Effective fertility data, season and rainfall blocks. (XLSX)

S4 Data Age and sex-structured sample counts of seven species of ungulates and ostrich along three road transects in Masai Mara National Reserve and its adjoining pastoral ranches (Koyiaki and Siana) from July 1989 to December 2003. (XLSX)

S5 Data Long-term rainfall in the Masai Mara National Reserve and in its neighbouring pastoral ranches (Koyiaki and Siana). (XLSX)

S1 File The monthly distribution of births among topi, warthog, hartebeest and impala in the Mara-Serengeti ecosystem, adapted from [44] (Fig A). Three adult, one yearling and one quarter-size topi, illustrating differences in body size, horn shape, horn size and body colour used to group the animals into size-classes. Photo credit: Niels Mogensen (Fig B).
(ZIP)

S2 File Relationships between a) early births (August-October), b) modal births (November-December), c) late births (January-March), e) annual fecundity and early rainfall (September-February); d) late births and late rainfall (March-June), f) annual fecundity and g) juvenile survival and annual rainfall (Nov-Jun); h) juvenile survival and early rainfall in the preceding year for warthog (Fig A). An adult female warthog with a quarter-size young (Fig B). Photo credit: Reto Buehler.
(ZIP)

S3 File Relationships between a) early births (August-October), b) modal births (November-December), c) late births (January-March), e) annual fecundity and early rainfall (September-February); d) late births and late rainfall (March-June), f) annual fecundity, g) neonate survival, i) juvenile survival, j) yearling survival and annual rainfall (Nov-Jun); k) neonate survival, juvenile survival and yearling survival and juvenile early rainfall in the preceding year for hartebeest (Fig A). One adult male and two three-quarter size male Coke’s hartebeests (Fig B). Photo credit: Niels Mogensen.
(ZIP)

S4 File Relationships between a) early births (August-October), b) modal births (November-December), c) late births (January-March), e) annual fecundity and early rainfall (September-February); d) late births and late rainfall (March-June), f) annual fecundity and g) juvenile survival and annual rainfall (Nov-Jun); h) juvenile survival and early rainfall for impala (Fig A). A full-grown and a young male impala, showing differences in horn size and shape used to group males into size classes (Fig B). Photo credit: Reto Buehler. A female impala in the company of three newborn lambs (Fig. C). Photo Credit: Reto Buehler.
(ZIP)

S1 Table Relationships between effective monthly fertility and the selected rainfall blocks spanning pre-conception months, grouped by season of conception. Significant effects are shown in bold face font.
(DOCX)

S2 Table Relationships between effective monthly fertility and selected rainfall blocks spanning pre-conception (Rain7_8 for topi and hartebeest and Rain6_7 for warthog and impala) and post-conception (Rain 9_11 for topi, Rain9_10 for hartebeest and Rain7_11 for warthog and impala) months grouped by season of conception. Significant effects are shown in bold face font.
(DOCX)

S3 Table Results of linear regression of early births in August-October (EbirthsAO), modal births in November-December (MbirthsND), late births in January-March (LbirthsJM), annual fecundity (Annfecund), neonatal (NeonAD), newborn (NJsurv), juvenile (Juven) and yearling (Yearl) survival on early rains spanning September-February (ErainsSF), late rains spanning March-June (LrainMJ) and annual rains covering September-October (AnRain) based on monthly ground counts conducted in the Masai Mara National Reserve from July 1989 to December 2002. Significant effects are shown in bold face font.
(DOCX)
