# Supplementary figures and images for: How Rainfall Variation Influences Reproductive Patterns of African Savanna Ungulates in an Equatorial Region Where Photoperiod Variation Is Absent
Source: PLoS One. 2015 Aug 21;10(8):e0133744. doi: 10.1371/journal.pone.0133744 (PMC4546645; doi:10.1371/journal.pone.0133744)

**Topi**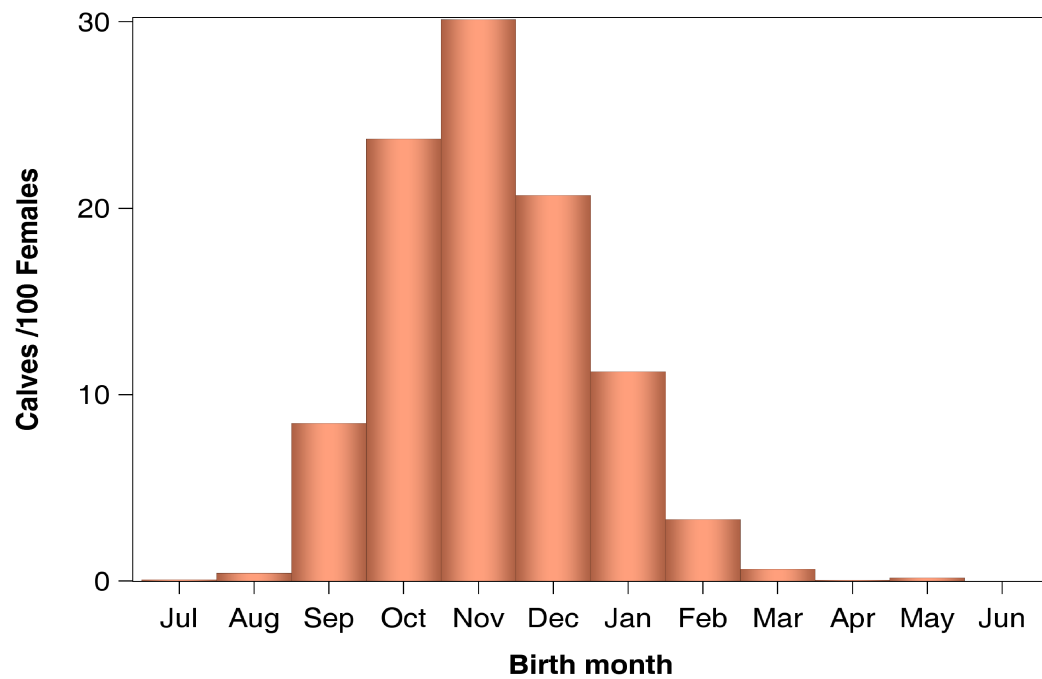**Hartebeest**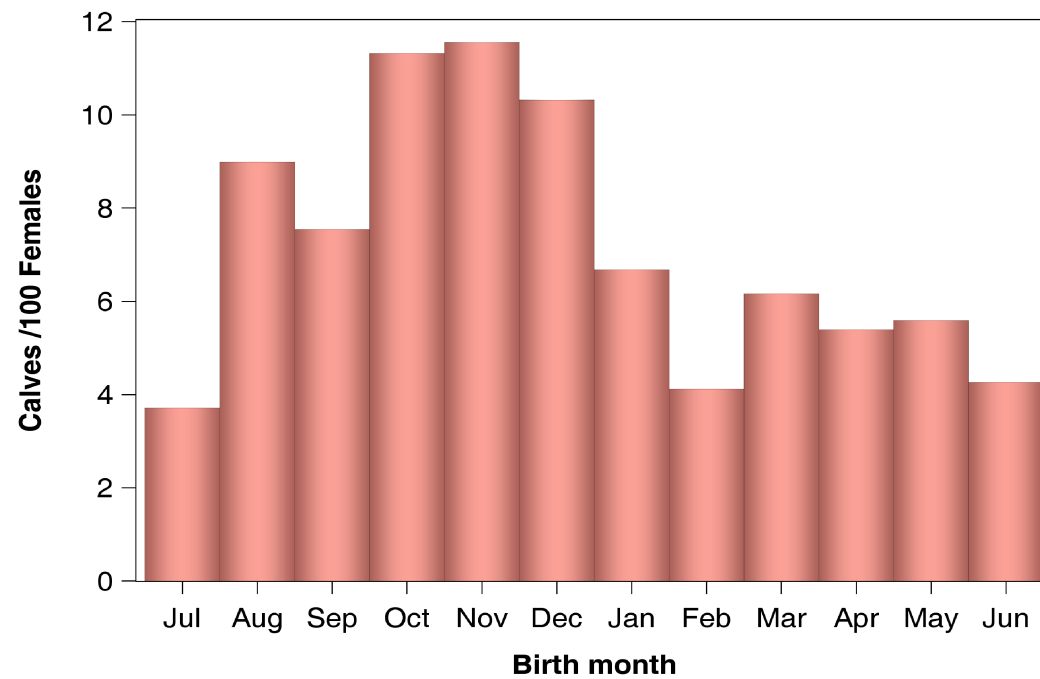**Warthog**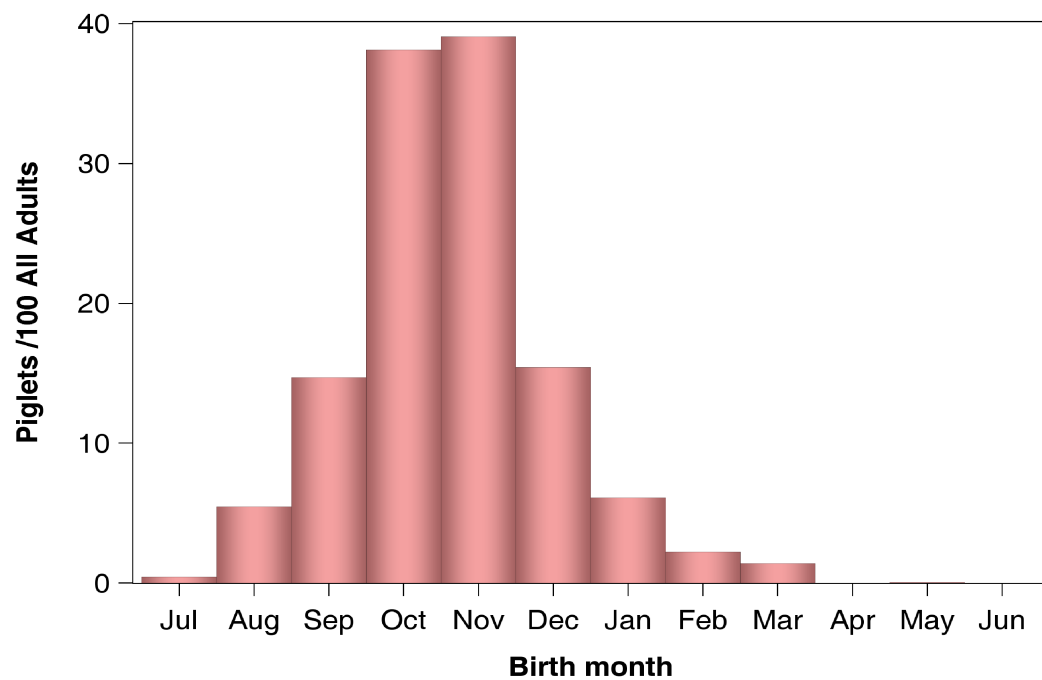**Impala**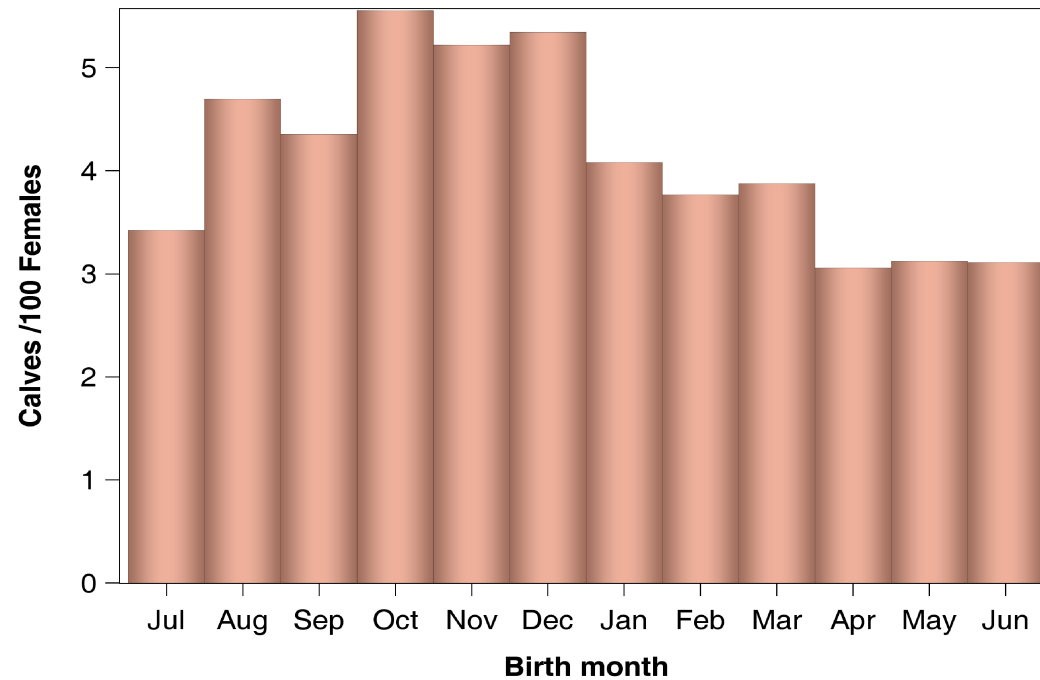

Supplement: S1 File — Three adult, one yearling and one quarter-size topi, illustrating differences in body size, horn shape, horn size and body colour used to group the animals into size-classes. Photo credit: Niels Mogensen (Fig B). (ZIP) [file pone.0133744.s006.zip › S1A Fig.pdf]

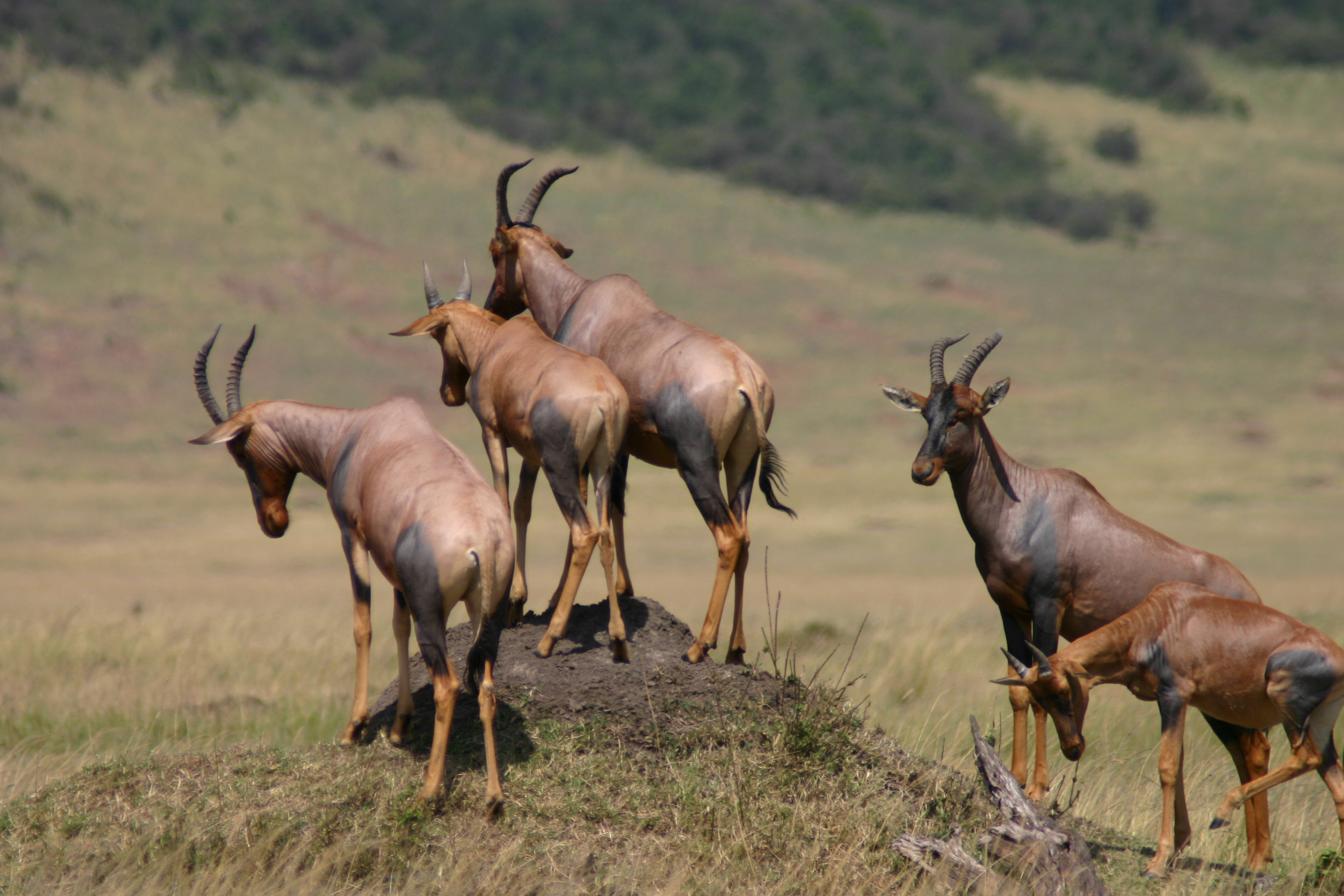

Supplement: S1 File — Three adult, one yearling and one quarter-size topi, illustrating differences in body size, horn shape, horn size and body colour used to group the animals into size-classes. Photo credit: Niels Mogensen (Fig B). (ZIP) [file pone.0133744.s006.zip › S1B Fig.jpg]

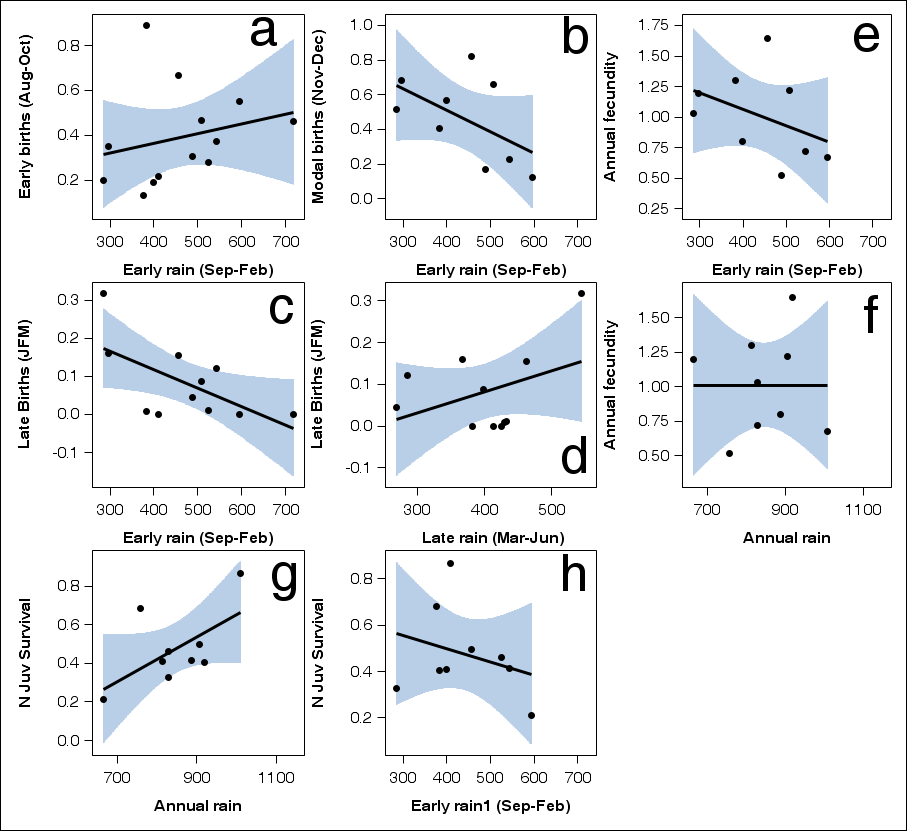

Supplement: S2 File — An adult female warthog with a quarter-size young (Fig B). Photo credit: Reto Buehler. (ZIP) [file pone.0133744.s007.zip › S2A Fig.png]

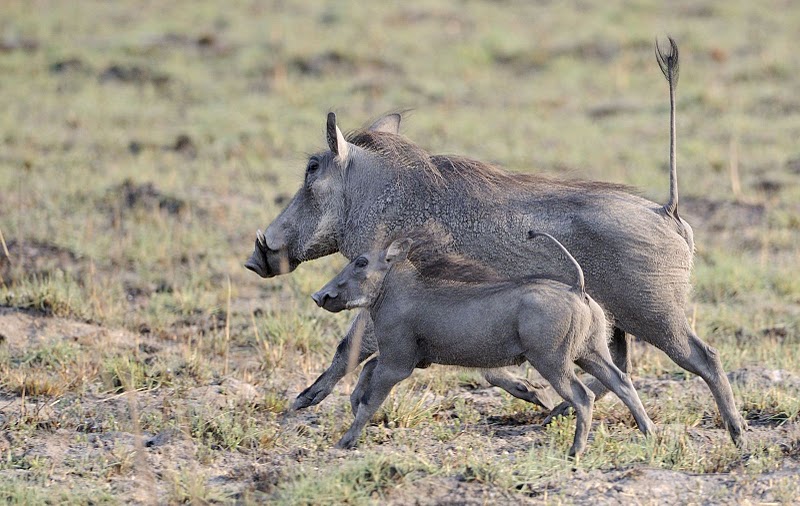

Supplement: S2 File — An adult female warthog with a quarter-size young (Fig B). Photo credit: Reto Buehler. (ZIP) [file pone.0133744.s007.zip › S2B Fig.jpg]

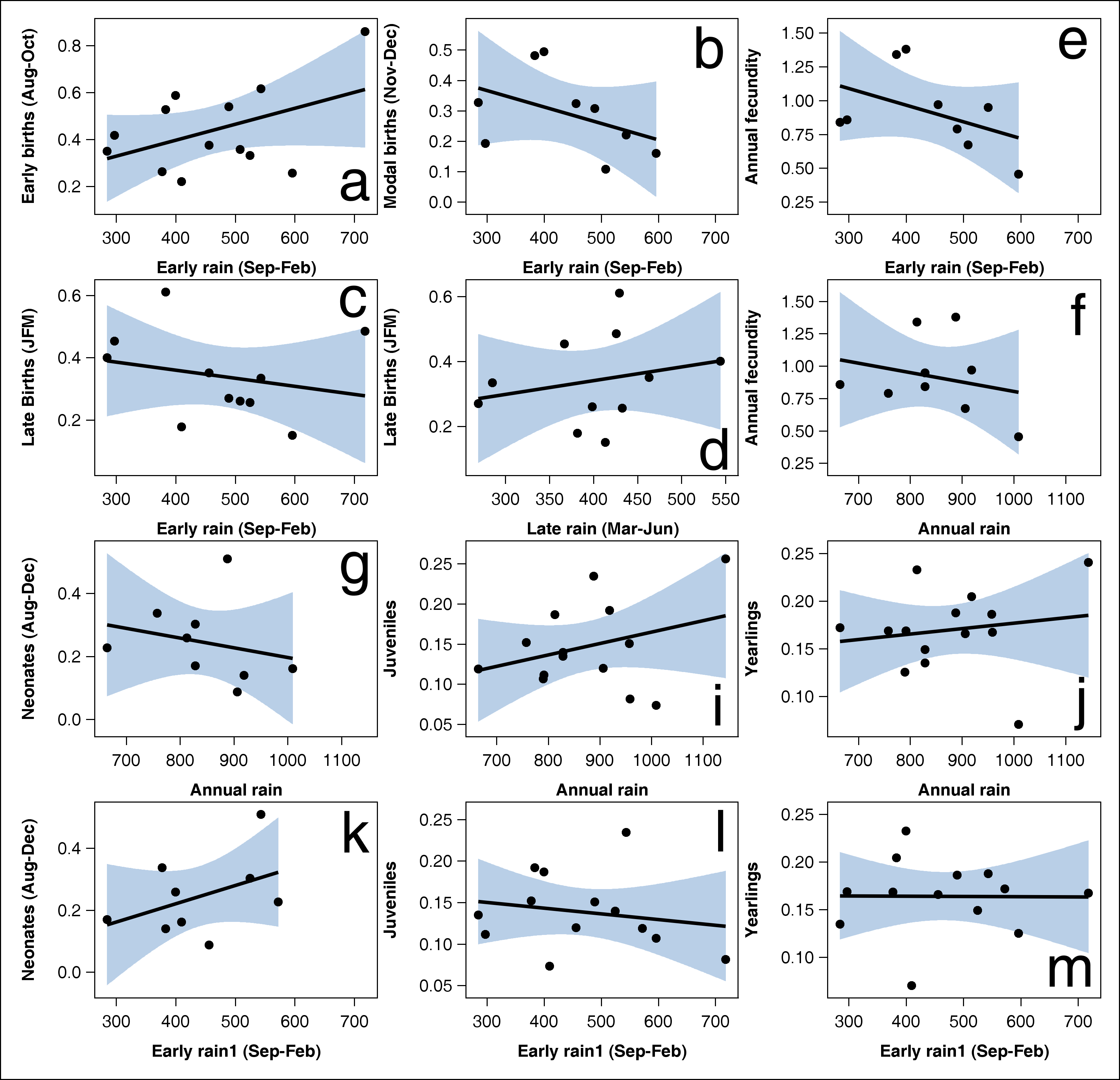

Supplement: S3 File — One adult male and two three-quarter size male Coke’s hartebeests (Fig B). Photo credit: Niels Mogensen. (ZIP) [file pone.0133744.s008.zip › S3A Fig.png]

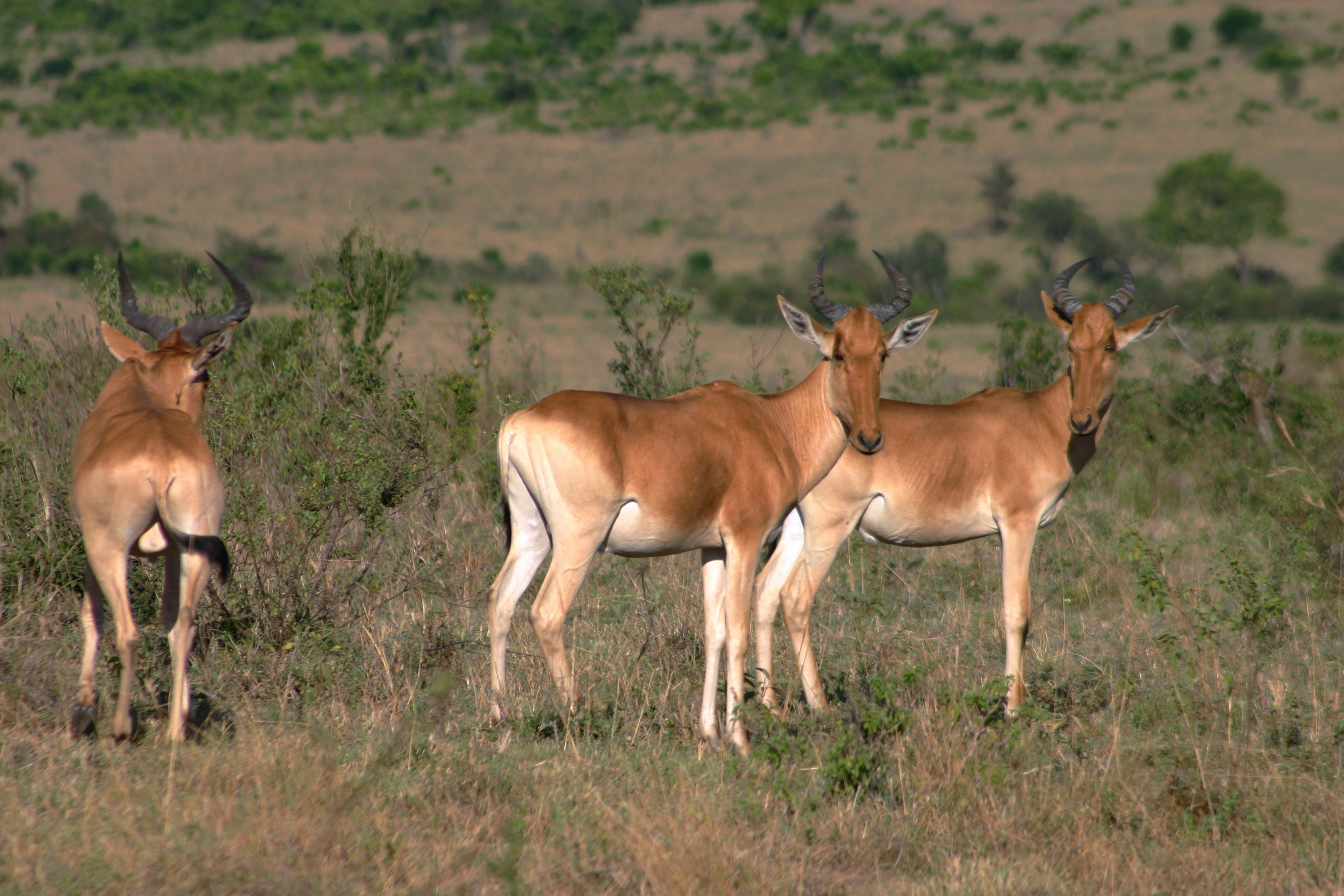

Supplement: S3 File — One adult male and two three-quarter size male Coke’s hartebeests (Fig B). Photo credit: Niels Mogensen. (ZIP) [file pone.0133744.s008.zip › S3B Fig.jpg]

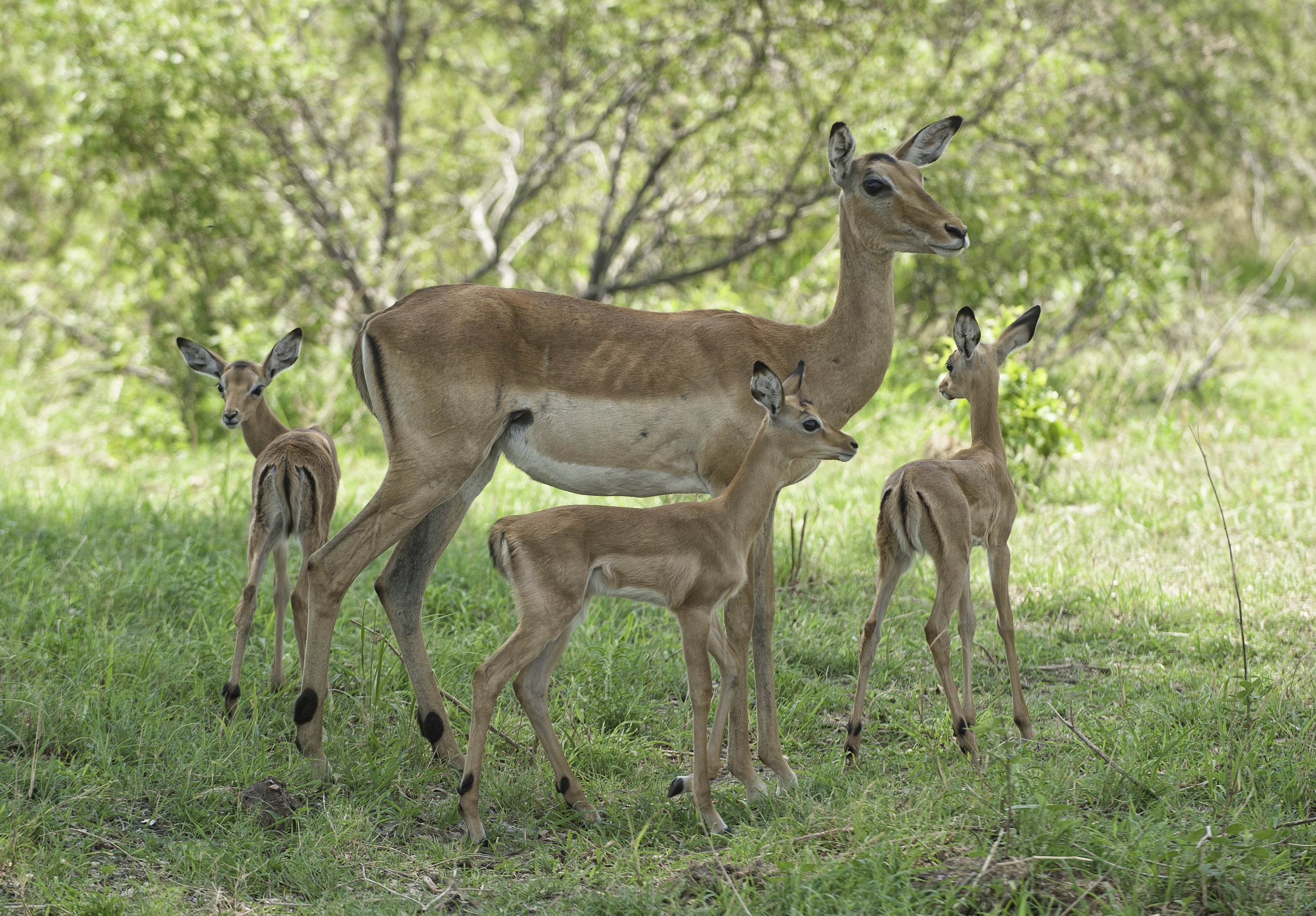

Supplement: S4 File — A full-grown and a young male impala, showing differences in horn size and shape used to group males into size classes (Fig B). Photo credit: Reto Buehler. A female impala in the company of three newborn lambs (Fig. C). Photo Credit: Reto Buehler. (ZIP) [file pone.0133744.s009.zip › S4C Fig.jpg]

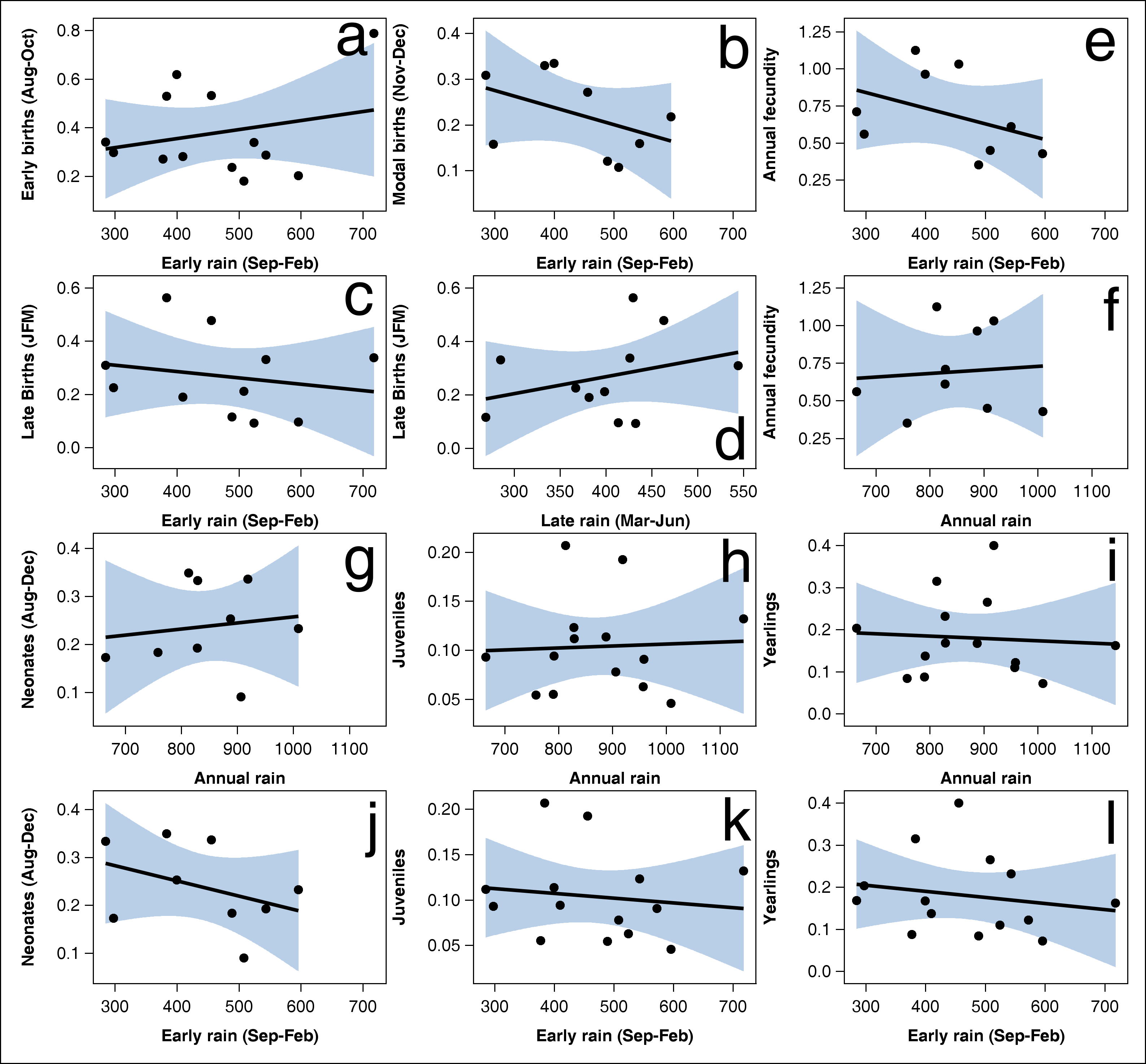

Supplement: S4 File — A full-grown and a young male impala, showing differences in horn size and shape used to group males into size classes (Fig B). Photo credit: Reto Buehler. A female impala in the company of three newborn lambs (Fig. C). Photo Credit: Reto Buehler. (ZIP) [file pone.0133744.s009.zip › S4A Fig.png]

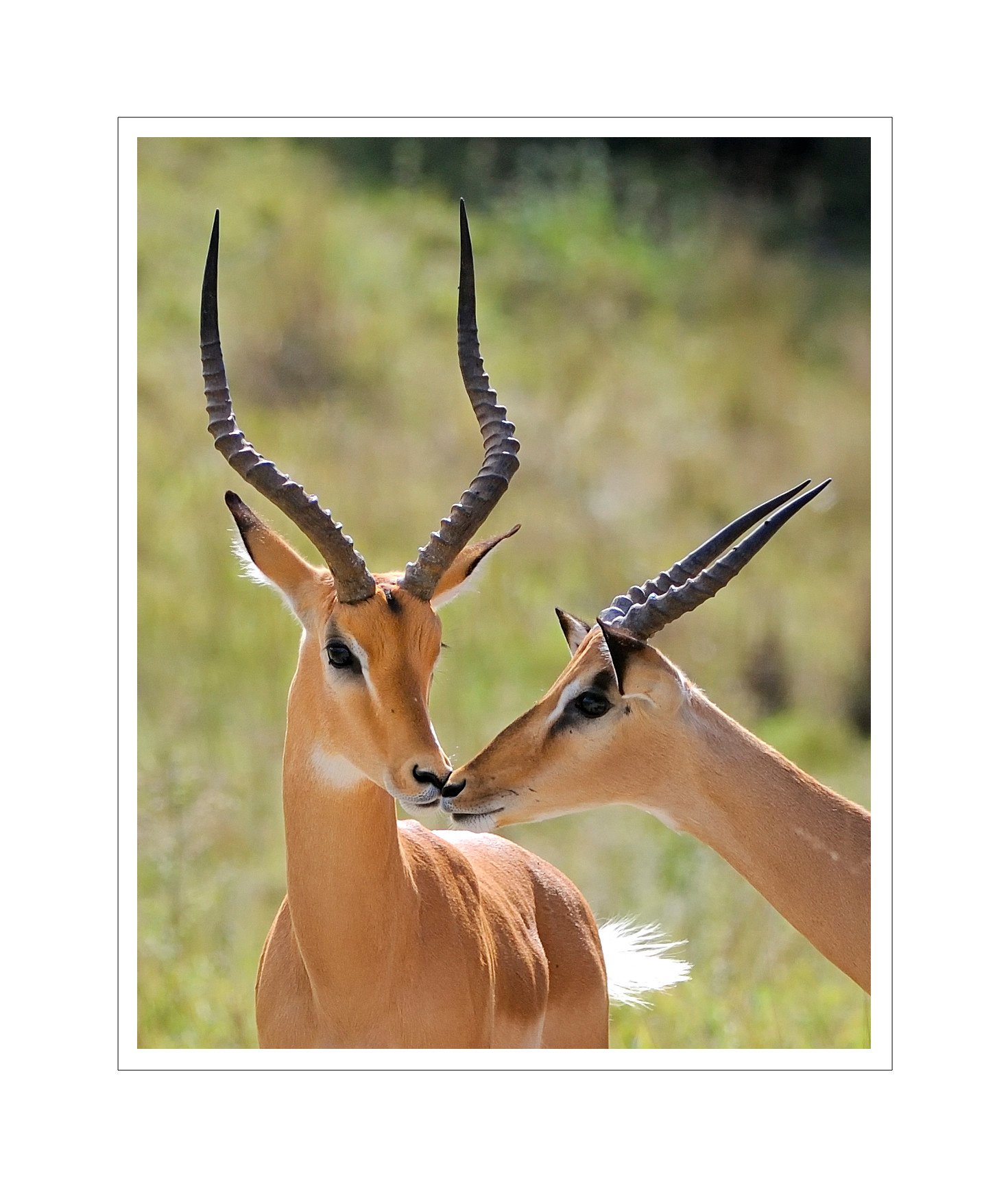

Supplement: S4 File — A full-grown and a young male impala, showing differences in horn size and shape used to group males into size classes (Fig B). Photo credit: Reto Buehler. A female impala in the company of three newborn lambs (Fig. C). Photo Credit: Reto Buehler. (ZIP) [file pone.0133744.s009.zip › S4B Fig.jpg]
