# Supplementary material for: How Rainfall Variation Influences Reproductive Patterns of African Savanna Ungulates in an Equatorial Region Where Photoperiod Variation Is Absent
Source: PLoS One. 2015 Aug 21;10(8):e0133744. doi: 10.1371/journal.pone.0133744 (PMC4546645; doi:10.1371/journal.pone.0133744)
Supplement: S1 Table — Significant effects are shown in bold face font. (DOCX) [file pone.0133744.s010.docx]

**S1 Table.** Relationships between effective monthly fertility and the selected rainfall blocks spanning pre-conception months, grouped by season of conception. Significant effects are shown in bold face font.

| **Species** | **Effect** | **Estimate** | **SE** | **DF** | **T** | **P>\|T\|** |
| --- | --- | --- | --- | --- | --- | --- |
| Topi | Intercept | -0.05035 | 0.052052 | 128 | -0.967 | 0.3352 |
|  | D×rain7_11 | 0.000159 | 0.000159 | 128 | 1.000 | 0.3193 |
|  | EW×rain7_11 | 0.000353 | 0.000134 | 128 | 2.630 | **0.0096** |
|  | LW×rain7_11 | 0.000528 | 0.000114 | 128 | 4.632 | **8.769 × 10^-6^** |
| Warthog | Intercept | -0.07298 | 0.070684 | 126 | -1.033 | 0.3038 |
|  | D×rain6_10 | 0.000265 | 0.000234 | 126 | 1.131 | 0.2601 |
|  | EW×rain6_10 | 0.000495 | 0.000164 | 126 | 3.013 | **0.0031** |
|  | LW×rain6_10 | 0.0006 | 0.00016 | 126 | 3.757 | **0.0003** |
| Hartebeest | Intercept | 0.038438 | 0.056643 | 127 | 0.679 | 0.4986 |
|  | D×rain7_10 | 0.000486 | 0.000251 | 127 | 1.935 | **0.0552** |
|  | EW×rain7_10 | 0.000323 | 0.000174 | 127 | 1.858 | **0.0655** |
|  | LW×rain7_10 | 0.000362 | 0.000155 | 127 | 2.340 | **0.0209** |
| Impala | Intercept | 0.028412 | 0.013439 | 128 | 2.114 | 0.0364 |
|  | D×rain6_10 | 0.000108 | 4.84×10^-5^ | 128 | 2.233 | **0.0273** |
|  | EW×rain6_10 | 6.99E-05 | 3.13×10^-5^ | 128 | 2.236 | **0.0271** |
|  | LW×rain6_10 | 9.1E-05 | 3.02×10^-5^ | 128 | 3.013 | **0.0031** |

^†^The numeric suffixes in rainfall blocks indicate the period in months over which moving averages of rainfall were computed. For example rain7_11 means that moving average of monthly rainfall was calculated over the 7^th^ to the 11^th^ month prior to the birth month. D=dry season, EW=Early wet season, LW=late wet season.
