# Supplementary material for: How Rainfall Variation Influences Reproductive Patterns of African Savanna Ungulates in an Equatorial Region Where Photoperiod Variation Is Absent
Source: PLoS One. 2015 Aug 21;10(8):e0133744. doi: 10.1371/journal.pone.0133744 (PMC4546645; doi:10.1371/journal.pone.0133744)
Supplement: S2 Table — Significant effects are shown in bold face font. (DOCX) [file pone.0133744.s011.docx]

**S2 Table.** Relationships between effective monthly fertility and selected rainfall blocks spanning pre-conception (Rain7_8 for topi and hartebeest and Rain6_7 for warthog and impala) and post-conception (Rain 9_11 for topi, Rain9_10 for hartebeest and Rain7_11 for warthog and impala) months grouped by season of conception. Significant effects are shown in bold face font.

| **Species** | **Effect^†^** | **Estimate** | **SE** | **DF** | **T** | **P>\|T\|** |
| --- | --- | --- | --- | --- | --- | --- |
| Topi | Intercept | -0.1285 | 0.0491 | 124 | -2.615 | 0.010036 |
|  | D×Rain7_8 | 0.0004 | 0.0004 | 124 | 1.137 | 0.257538 |
|  | EW×Rain7_8 | -0.0001 | 0.0002 | 124 | -0.260 | 0.795308 |
|  | LW×Rain7_8 | 0.0004 | 0.0002 | 124 | 1.869 | **0.06394** |
|  | D×Rain9_11 | 0.0003 | 0.0002 | 124 | 1.697 | **0.09228** |
|  | EW×Rain9_11 | 0.0011 | 0.0002 | 124 | 5.104 | **1.2 × 10^-6^** |
|  | LW×Rain9_11 | 0.0028 | 0.0006 | 124 | 4.850 | **3.6 × 10^-6^** |
|  | LW×Rain9×Rain9_11 | 0.0000 | 0.0000 | 124 | -4.724 | **6.2 × 10^-6^** |
|  |  |  |  |  |  |  |
| Warthog | Intercept | -0.0564 | 0.0699 | 123 | -0.806 | 0.421926 |
|  | D×Rain6_7 | 0.0001 | 0.0004 | 123 | 0.251 | 0.802179 |
|  | EW×Rain6_7 | 0.0001 | 0.0003 | 123 | 0.448 | 0.654626 |
|  | LW×Rain6_7 | 0.0014 | 0.0004 | 123 | 3.886 | **0.00017** |
|  | D×Rain7_10 | 0.0003 | 0.0004 | 123 | 0.795 | 0.428032 |
|  | EW×Rain7_10 | 0.0007 | 0.0003 | 123 | 2.695 | **0.00802** |
|  | LW×Rain7_10 | 0.0002 | 0.0002 | 123 | 0.644 | 0.520814 |
|  |  |  |  |  |  |  |
| Hartebeest | Intercept | 0.1547 | 0.0629 | 123 | 2.459 | 0.015306 |
|  | D×Rain7_8 | -0.0003 | 0.0006 | 123 | -0.605 | 0.546192 |
|  | EW×Rain7_8 | 0.0001 | 0.0003 | 123 | 0.507 | 0.613309 |
|  | LW×Rain7_8 | 0.0005 | 0.0003 | 123 | 1.819 | **0.07138** |
|  | D×Rain9_10 | 0.0003 | 0.0004 | 123 | 0.764 | 0.446157 |
|  | EW×Rain9_10 | -0.0018 | 0.0009 | 123 | -2.045 | **0.04303** |
|  | EW×Rain9_10×Rain9_10 | 0.0000 | 0.0000 | 123 | 3.331 | **0.00114** |
|  | LW×Rain9_10 | -0.0003 | 0.0003 | 123 | -1.159 | 0.248709 |
|  |  |  |  |  |  |  |
| Impala | Intercept | 0.0454 | 0.0223 | 122 | 2.039 | 0.043638 |
|  | D×Rain6_7 | -0.0001 | 0.0001 | 122 | -0.614 | 0.54063 |
|  | EW×Rain6_7 | 0.0002 | 0.0002 | 122 | 0.668 | 0.505251 |
|  | EW×Rain6_7×Rain6_7 | 0.0000 | 0.0000 | 122 |  | **0** |
|  | LW×Rain6_7 | 0.0001 | 0.0001 | 122 | 2.150 | 0.033555 |
|  | D×Rain7_10 | 0.0001 | 0.0001 | 122 | 1.370 | 0.173352 |
|  | EW×Rain7_10 | -0.0001 | 0.0002 | 122 | -0.750 | 0.454539 |
|  | EW×Rain7_10×Rain7_10 | 0.0000 | 0.0000 | 122 |  | **0** |
|  | LW×Rain7_10 | -0.0001 | 0.0002 | 122 | -0.623 | 0.534164 |
|  | LW×Rain7_10×Rain7_10 | 0.0000 | 0.0000 | 122 |  | **0** |
|  |  |  |  |  |  |  |

^†^The numeric suffixes in rainfall blocks indicate the period in months over which moving averages of rainfall were computed. For example rain7_11 means that moving average of monthly rainfall was calculated over the 7^th^ to the 11^th^ month prior to the birth month. D=dry season, EW=Early wet season, LW=late wet season.
