# Supplementary material for: How Rainfall Variation Influences Reproductive Patterns of African Savanna Ungulates in an Equatorial Region Where Photoperiod Variation Is Absent
Source: PLoS One. 2015 Aug 21;10(8):e0133744. doi: 10.1371/journal.pone.0133744 (PMC4546645; doi:10.1371/journal.pone.0133744)
Supplement: S3 Table — Significant effects are shown in bold face font. (DOCX) [file pone.0133744.s012.docx]

**S3 Table.** Results of linear regression of early births in August-October (EbirthsAO), modal births in November-December (MbirthsND), late births in January-March (LbirthsJM), annual fecundity (Annfecund), neonatal (NeonAD), newborn (NJsurv), juvenile (Juven) and yearling (Yearl) survival on early rains spanning September-February (ErainsSF), late rains spanning March-June (LrainMJ) and annual rains covering September-October (AnRain) based on monthly ground counts conducted in the Masai Mara National Reserve from July 1989 to December 2002. Significant effects are shown in bold face font.

| **Species** | **Response** | **Effect** | **Estimate** | **SE** | **DF** | **T** | **P>\|T\|** |
| --- | --- | --- | --- | --- | --- | --- | --- |
| Hartebeest | EbirthsAO | Intercept | 0.1252 | 0.1903 | 11 | 0.658 | 0.5241 |
|  |  | ErainSF | 0.0007 | 0.0004 | 11 | 1.700 | 0.1171 |
| Impala | EbirthsAO | Intercept | 0.2068 | 0.2097 | 11 | 0.986 | 0.3452 |
|  |  | ErainSF | 0.0004 | 0.0004 | 11 | 0.840 | 0.4188 |
| Topi | EbirthsAO | Intercept | -0.3710 | 0.1025 | 11 | -3.619 | 0.0040 |
|  |  | ErainSF | 0.0012 | 0.0002 | 11 | 5.738 | **0.0001** |
| Warthog | EbirthsAO | Intercept | 0.1913 | 0.2472 | 11 | 0.774 | 0.4552 |
|  |  | ErainSF | 0.0004 | 0.0005 | 11 | 0.832 | 0.4232 |
| Hartebeest | MbirthsND | Intercept | 0.5284 | 0.1942 | 7 | 2.721 | 0.0297 |
|  |  | ErainSF | -0.0005 | 0.0004 | 7 | -1.254 | 0.2500 |
| Impala | MbirthsND | Intercept | 0.3866 | 0.1299 | 7 | 2.977 | 0.0206 |
|  |  | ErainSF | -0.0004 | 0.0003 | 7 | -1.292 | 0.2372 |
| Topi | MbirthsND | Intercept | 0.8628 | 0.3083 | 8 | 2.798 | 0.0233 |
|  |  | ErainSF | -0.0011 | 0.0007 | 8 | -1.635 | 0.1407 |
| Warthog | MbirthsND | Intercept | 1.0092 | 0.3352 | 7 | 3.010 | 0.0196 |
|  |  | ErainSF | -0.0012 | 0.0007 | 7 | -1.671 | 0.1387 |
| Hartebeest | LbirthJM | Intercept | 0.4637 | 0.1715 | 9 | 2.704 | 0.0242 |
|  |  | ErainSF | -0.0003 | 0.0004 | 9 | -0.737 | 0.4800 |
| Impala | LbirthJM | Intercept | 0.3810 | 0.1924 | 9 | 1.981 | 0.0789 |
|  |  | ErainSF | -0.0002 | 0.0004 | 9 | -0.605 | 0.5604 |
| Topi | LbirthJM | Intercept | 0.2719 | 0.1139 | 9 | 2.387 | 0.0408 |
|  |  | ErainSF | -0.0004 | 0.0002 | 9 | -1.665 | 0.1302 |
| Warthog | LbirthJM | Intercept | 0.3126 | 0.1008 | 9 | 3.101 | 0.0127 |
|  |  | ErainSF | -0.0005 | 0.0002 | 9 | -2.359 | **0.0427** |
| Hartebeest | LbirthJM | Intercept | 0.1712 | 0.2369 | 9 | 0.723 | 0.4883 |
|  |  | LrainMJ | 0.0004 | 0.0006 | 9 | 0.730 | 0.4841 |
| Impala | LbirthJM | Intercept | 0.0151 | 0.2570 | 9 | 0.059 | 0.9544 |
|  |  | LrainMJ | 0.0006 | 0.0006 | 9 | 1.002 | 0.3426 |
| Topi | LbirthJM | Intercept | 0.2034 | 0.1757 | 9 | 1.158 | 0.2768 |
|  |  | LrainMJ | -0.0003 | 0.0004 | 9 | -0.667 | 0.5217 |
| Warthog | LbirthJM | Intercept | -0.1215 | 0.1631 | 9 | -0.745 | 0.4753 |
|  |  | LrainMJ | 0.0005 | 0.0004 | 9 | 1.271 | 0.2357 |
| Hartebeest | Annfecund | Intercept | 1.4594 | 0.4201 | 7 | 3.474 | 0.0103 |
|  |  | ErainSF | -0.0012 | 0.0009 | 7 | -1.323 | 0.2273 |
| Impala | Annfecund | Intercept | 1.1562 | 0.4150 | 7 | 2.786 | 0.0271 |
|  |  | ErainSF | -0.0011 | 0.0009 | 7 | -1.145 | 0.2898 |
| Topi | Annfecund | Intercept | 0.8045 | 0.3067 | 8 | 2.623 | 0.0305 |
|  |  | ErainSF | -0.0003 | 0.0007 | 8 | -0.445 | 0.6681 |
| Warthog | Annfecund | Intercept | 1.5878 | 0.5315 | 7 | 2.987 | 0.0203 |
|  |  | ErainSF | -0.0013 | 0.0012 | 7 | -1.115 | 0.3015 |
| Hartebeest | Annfecund | Intercept | 1.5314 | 0.9205 | 7 | 1.664 | 0.1401 |
|  |  | AnRain | -0.0007 | 0.0011 | 7 | -0.671 | 0.5238 |
| Impala | Annfecund | Intercept | 0.4876 | 0.9110 | 7 | 0.535 | 0.6090 |
|  |  | AnRain | 0.0002 | 0.0011 | 7 | 0.227 | 0.8271 |
| Topi | Annfecund | Intercept | 0.5432 | 0.6044 | 8 | 0.899 | 0.3950 |
|  |  | AnRain | 0.0001 | 0.0007 | 8 | 0.213 | 0.8365 |
| Warthog | Annfecund | Intercept | 1.0148 | 1.1663 | 7 | 0.870 | 0.4131 |
|  |  | AnRain | 0.0000 | 0.0014 | 7 | -0.004 | 0.9968 |
| Hartebeest | NeonAD | Intercept | 0.5048 | 0.4001 | 7 | 1.262 | 0.2475 |
|  |  | AnRain | -0.0003 | 0.0005 | 7 | -0.656 | 0.5330 |
| Impala | NeonAD | Intercept | 0.1314 | 0.2814 | 7 | 0.467 | 0.6547 |
|  |  | AnRain | 0.0001 | 0.0003 | 7 | 0.382 | 0.7135 |
| Hartebeest | NeonAD | Intercept | -0.0161 | 0.2097 | 7 | -0.077 | 0.9411 |
|  |  | ErainSF1 | 0.0006 | 0.0005 | 7 | 1.265 | 0.2463 |
| Impala | NeonAD | Intercept | 0.3780 | 0.1298 | 7 | 2.912 | 0.0226 |
|  |  | ErainSF | -0.0003 | 0.0003 | 7 | -1.104 | 0.3062 |
| Topi | NJsurv | Intercept | -0.2708 | 0.3232 | 8 | -0.838 | 0.4264 |
|  |  | AnRain | 0.0008 | 0.0004 | 8 | 2.221 | **0.0571** |
| Warthog | NJsurv | Intercept | -0.5023 | 0.5036 | 7 | -0.997 | 0.3518 |
|  |  | AnRain | 0.0012 | 0.0006 | 7 | 1.952 | **0.0918** |
| Topi | NJsurv | Intercept | 0.2164 | 0.2200 | 8 | 0.984 | 0.3540 |
|  |  | ErainSF1 | 0.0005 | 0.0005 | 8 | 1.048 | 0.3253 |
| Warthog | NJsurv | Intercept | 0.7250 | 0.3268 | 7 | 2.218 | 0.0620 |
|  |  | ErainSF1 | -0.0006 | 0.0007 | 7 | -0.781 | 0.4603 |
| Topi | NJsurv | Intercept | 0.2203 | 0.1381 | 8 | 1.595 | 0.1493 |
|  |  | Dry1 | 0.0011 | 0.0006 | 8 | 1.677 | 0.1320 |
| Warthog | NJsurv | Intercept | 0.0620 | 0.1741 | 7 | 0.356 | 0.7325 |
|  |  | Dry1 | 0.0019 | 0.0008 | 7 | 2.479 | **0.0423** |
| Topi | NYsurv | Intercept | 0.0144 | 0.1229 | 8 | 0.118 | 0.9094 |
|  |  | ErainSF1 | 0.0005 | 0.0003 | 8 | 1.808 | 0.1082 |
| Topi | NYsurv | Intercept | 0.1468 | 0.0947 | 8 | 1.550 | 0.1598 |
|  |  | Dry1 | 0.0004 | 0.0004 | 8 | 0.943 | 0.3734 |
| Hartebeest | Juven | Intercept | 0.0229 | 0.1080 | 12 | 0.212 | 0.8358 |
|  |  | AnRain | 0.0001 | 0.0001 | 12 | 1.161 | 0.2681 |
| Impala | Juven | Intercept | 0.0860 | 0.1030 | 12 | 0.836 | 0.4198 |
|  |  | AnRain | 0.0000 | 0.0001 | 12 | 0.175 | 0.8637 |
| Hartebeest | Juven | Intercept | 0.1705 | 0.0519 | 11 | 3.284 | 0.0073 |
|  |  | ErainSF1 | -0.0001 | 0.0001 | 11 | -0.629 | 0.5419 |
| Impala | Juven | Intercept | 0.1285 | 0.0559 | 12 | 2.297 | 0.0405 |
|  |  | ErainSF | -0.0001 | 0.0001 | 12 | -0.452 | 0.6594 |
| Hartebeest | Yearl | Intercept | 0.1198 | 0.0906 | 12 | 1.323 | 0.2106 |
|  |  | AnRain | 0.0001 | 0.0001 | 12 | 0.555 | 0.5892 |
| Impala | Yearl | Intercept | 0.2290 | 0.2011 | 12 | 1.139 | 0.2770 |
|  |  | AnRain | -0.0001 | 0.0002 | 12 | -0.242 | 0.8129 |
| Hartebeest | Yearl | Intercept | 0.1612 | 0.0501 | 12 | 3.215 | 0.0074 |
|  |  | ErainSF1 | 0.0000 | 0.0001 | 12 | 0.173 | 0.8657 |
| Impala | Yearl | Intercept | 0.2488 | 0.1084 | 12 | 2.295 | 0.0406 |
|  |  | ErainSF | -0.0001 | 0.0002 | 12 | -0.647 | 0.5299 |
